# Periodontitis and Helicobacter pylori Infection: Eradication and Periodontal Therapy Combination

**DOI:** 10.1055/s-0041-1731928

**Published:** 2021-10-01

**Authors:** Athanasios Tsimpiris, Andreas Grigoriadis, Ioannis Tsolianos, Ioannis Moschos, Dimitrios G. Goulis, Georgios Kouklakis

**Affiliations:** 1 Department of Medicine, Democritus University of Thrace, Alexandroupolis, Greece; 2 Dental Sector, 424 General Military Training Hospital, Thessaloniki, Greece; 3 Department of Preventive Dentistry, Periodontology and Implant Biology, Dental School, Faculty of Health Sciences, Aristotle University of Thessaloniki, Thessaloniki, Greece; 4 Dental School, Faculty of Health Sciences, Aristotle University of Thessaloniki, Thessaloniki, Greece; 5 Department of Nursing, International Hellenic University, Thessaloniki, Greece; 6 Unit of Reproductive Endocrinology, First Department of Obstetrics and Gynecology, Medical School, Aristotle University of Thessaloniki, Thessaloniki, Greece; 7 A' Department of Pathology, Department of Medicine, Democritus University of Thrace, Alexandroupolis, Greece

**Keywords:** *Helicobacter pylori*, periodontitis, saliva, oral cavity, eradication, periodontal therapy

## Abstract

**Objectives** This study was aimed to assess (1) the prevalence of salivary and gastric *Helicobacter pylori* (HP) infection in patients with and without periodontitis, (2) the prevalence of HP infection in patients with periodontitis according to its clinical classification, (3) the effect of periodontitis treatment in patients with or without gastric HP infection, and (4) if gastric HP eradication in combination with periodontitis treatment influences periodontitis clinical outcome.

**Materials and Methods** Thirty-three adults with periodontitis treated by quadrant scaling and root planning (QSRP). The simplified plaque index (PI), bleeding on probing index (BOP), probing pocket depth (PPD), and clinical attachment level (CAL) were assessed pretreatment and 3 months of posttreatment. The patients were tested for oral and gastric HP. Gastric HP (+) patients received eradication therapy. A control group of 32 periodontically healthy volunteers was tested for oral and gastric HP. Saliva samples were evaluated by real-time polymerase chain reaction (rtPCR); gastric HP was detected by urea breath test (UBT).

**Statistical Analysis** Normality of variables assessed by the Kolmogorov–Smirnov test, while the differences of pre- and post-treatment were analyzed by paired samples *t* -test. Differences between subgroups were compared by a Student’s *t* -test or a Mann–Whitney *U* -test. Comparisons of nominal variables were made by Pearson’s Chi-square test.

**Results** No saliva samples were positive for HP. Gastric HP was detected in six patients with periodontitis and seven controls ( *p* > 0.05). HP infection affected patients with higher disease stages and grades. All HP (+) patients underwent successful eradication treatment. All clinical periodontitis indices improved following QSRP. HP (+) patients who received QSRP as adjunctive to eradication treatment showed improvement in BOP ( *p* < 0.001), PI ( *p* < 0.013), and CAL ( *p* < 0.004) compared with HP (−) patients who received QSRP alone.

**Conclusion** Periodontitis was not associated with gastric HP infection. Saliva was not a gastric HP supply reservoir. Gastric HP infection was associated with advanced stages and grades of periodontitis. Although all periodontal clinical markers improved after QSRP, BOP, PI, and CAL, they were further improved when combined with eradication treatment. Periodontal evaluation and treatment combined with HP eradication are recommended in patients with HP gastric infection.

## Introduction

Advanced periodontitis is the sixth commonest disease worldwide, with a prevalence of 10.8 to 11.2%. 1 2 It is a chronic inflammatory disease of the periodontal tissues due to anaerobic gram-negative bacteria 3 that is characterized by progressive alveolar bone destruction, resulting in the formation of a periodontal pocket and gum retraction. 4 Periodontal disease can be considered to disrupt homeostatic mechanisms that balance the oral biofilm microflora and the host defense mechanisms. 5 Periodontitis has been associated with cardiovascular disease, certain types of cancer, type-2 diabetes, and pregnancy complications. 6

*Helicobacter pylori* (HP) is a microaerophilic gram-negative spiral-helical bacterium adapted to survive in the gastric acidic environment, neutralizing it by secreting urease. 7 A higher prevalence of gastric HP infection exists in developing countries (50.8%) compared with the developed ones (34.7%). 8 Gastric mucosal infection by HP has been associated with gastritis, gastric and duodenal ulcers, and gastric cancer. 9 In addition, HP infection has been associated with several nonpeptic diseases, such as coronary heart disease and cardiovascular disease, diabetes mellitus, and anemia. 10 Also, HP infection has been associated with oral cavity diseases, such as recurrent aphthous stomatitis, 11 halitosis, burning mouth syndrome, lingual hyperplasia, 12 and oral lichen planus. 13

Treatment of HP infection is by combinations of antibiotics and proton pump inhibitors (PPI), taken simultaneously or sequentially for 7 to 14 days; however, in clinical practice, no treatment guarantees HP eradication. 14 The high rates of HP infection recurrence led researchers to investigate whether the oral cavity is an extra-gastric HP reservoir, as systemic antimicrobials against HP fail to disrupt the germ-protective oral biofilm. 15 Studies demonstrated the permissive role of chronic periodontal disease in the colonization of the oral cavity and gastric infection by HP. 16

The aims of the present study were (1) to assess the prevalence of HP infection in patients with and without periodontitis; (2) to assess the prevalence of HP infection in patients with periodontitis according to the clinical classification of the latter; (3) to evaluate the effect of periodontitis treatment in patients with or without gastric HP infection; and (4) to evaluate if gastric HP eradication treatment in patients with HP infection treatment in combination with periodontitis treatment influences periodontitis clinical outcome.

## Materials and Methods

### Patients

Thirty-three patients attended the hospital’s dental sector from April 2019 to October 2020 and were diagnosed with periodontitis stage ≥2. 17 Thirty-two periodontically healthy volunteers, matched for sex and age to the patients, served as a control group. Exclusion criteria were age <18 years, previous HP eradication attempt, use of antibiotics, bismuth compounds, PPIs, H _2_ blockers, or antacids within the last 2 months, pregnancy, diabetes, immune diseases, immunosuppression for any cause, chronic use of nonsteroidal anti-inflammatory drugs (NSAIDs), history of gastric surgery, malignancies, periodontal treatment in the last 6 months, and <18 natural teeth.

### Protocol

The periodontal examination was performed at six sites of each tooth (mesiobuccal, midbuccal, distobuccal, mesiolingual, midlingual, and distolingual) and included for each site as follows: probing pocket depth (PPD; distance from the gingival margin to the bottom of the gingival sulcus, in mm), evaluation of clinical attachment level (CAL; distance from the cementoenamel junction to the bottom of the gingival sulcus, in mm), simplified plaque index (PI; presence or absence of supragingival plaque using basic fuchsin as a disclosing agent), and presence or absence of bleeding on probing (BOP) 30 seconds after PPD. An orthopantomography was performed in all patients for radiographic evaluation of the jawbone. Patients with PPD ≥ 4 mm and/or CAL ≥ 4 mm >30% of the measurement surfaces and BOP > 10% were classified into the test group ( *n* = 33), while those with BOP < 10% and PPD ≤ 3 mm were classified into the control group ( *n* = 32). All measurements were made using a manual periodontal probe (University of North Carolina - 15, Hu-Friedy) by the same experienced periodontist (A.G.). The intraexaminer error was high (kappa > 0.80), and the measurements had >90% agreement for ± 1 mm and exact agreement in >75% of the PPD repeated measurements. 18 The presence of HP in saliva and stomach in both groups was detected by real-time polymerase chain reaction (rtPCR) and urea breath test (UBT) method, respectively.

During a second visit, a saliva sample was collected, and the detection of gastric HP was performed by UBT. All patients arrived in the morning without eating for >6 hours and without brushing their teeth or rinsing their mouths. The saliva was collected by a sterile swab with a synthetic fiber tip to wipe the inside of the cheeks and placed in a sterile 2-ml Eppendorf with 500 μL NaCl 0.9%; it was stored at 2 to 8°C for 18 to 24 hours or at −20°C for up to 3 months, depending on the test time.

### Real-Time Polymerase Chain Reaction

The VIASURE HP rtPCR Detection Kit (CERTEST BIOTEC, Spain, CE-IVD) was used for the molecular detection of HP which was evaluated against strain J99, targeting in virulence gen ureB that encodes one of bacterial urease’s structural subunit. DNA extraction was performed by the PREP-NADNA/RNA Extraction Kit, DNA Technology, CE-IVD. The VIASURE kit is based on the five-exonuclease activity of DNA polymerase. The fluorescence was measured by Stratagene mx3005p (Agilent, United States).

### Urea Breath Test

The *Helicobacter* test INFAI 75 mg ^13^ C was used. Two breath samples were collected by blowing through a straw into two glass tubes with a stopper. Subsequently, each patient received 200 mL of natural orange juice to delay gastric emptying and ingested a drink containing 75 mg ^13^ C-marked urea (30 mL). After 30 minutes, the blowing exercise was repeated to collect postdose samples. The presence of gastric HP leads to hydrolysis of urea by the enzyme urease and release of marked ^13^ CO _2_ . The four breath samples were sent to a central laboratory for ^13^ C/ ^12^ C ratio analyses on exhaled CO _2_ by mass spectrometry. HP infection was detected if the difference in ^13^ C/ ^12^ C ratio between the pre- and post-C samples were >4.0%. UBT presents high sensitivity (>95%) and specificity (>93%). 19

### Treatment

**Fig. 1:**
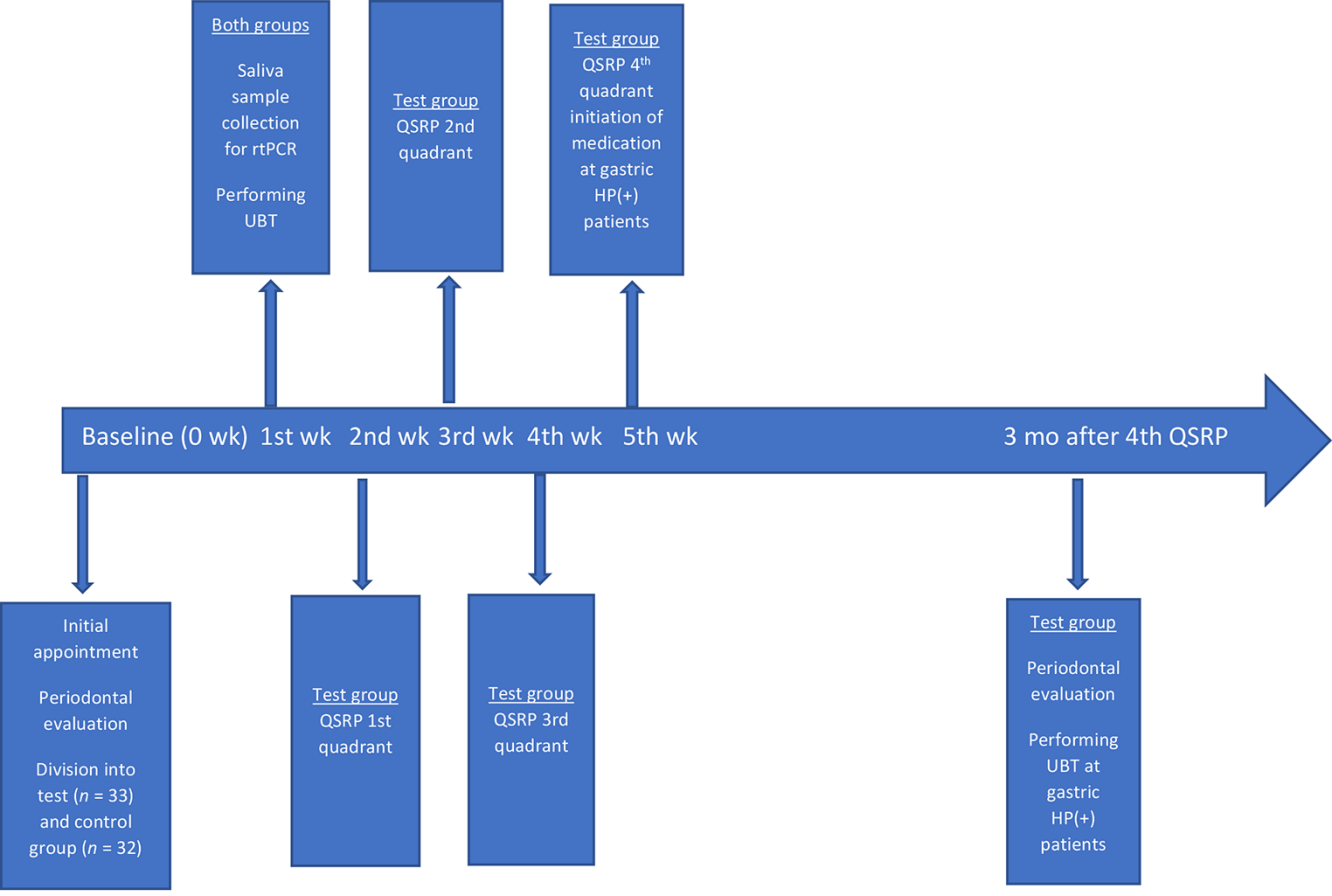
Timeline and study protocol diagram. HP, *Helicobacter pylori* ; QSRP, quadrant scaling and root planning; rtPCR, real-time polymerase chain reaction; UBT, urea breath test.

All patients in the test group were treated with quadrant scaling and root planning (QSRP) under local anesthesia (4% articaine hydrochloride with epinephrine 1:100,000) in four appointments, with an interval of 1 week between them and reexamined after 3 months. The instruments used were periodontal curettes (Gracey Access curettes, Kohler, Austria) and ultrasonic scalers (Piezon 250, EMS, Switzerland). In gastric HP (+) patients, sequential therapy was administered for 10 days (dual therapy including a PPI plus amoxicillin 1-g twice daily both, for the first 5 days followed by a triple therapy including a PPI, clarithromycin of 500 mg and metronidazole of 500-mg twice daily all, for the remaining 5 days) immediately after the end of QSRP. Three months after QSRP, measurements of clinical periodontal markers were performed by the same periodontist who performed the initial examination, not knowing which patients received antibiotic treatment and by the same manual periodontal probe. The efficacy of gastric HP eradication in HP (+) patients from the experimental group was also tested by UBT, 3 months after QSRP. The study protocol and timeline diagram it is shown in **Fig. 1** .

### Preliminary Power Analysis

As a preliminary power test, assuming a mean PPD difference of 2 mm with a pooled standard deviation of 1 mm, we would require six patients in our gastric HP (+) sample to achieve power exceeding the 0.8 rule-of-thumb (0.876). 20

### Statistical Methods

The normality of variables (BOP, PI, PPD, and CAL) was assessed by the Kolmogorov–Smirnov test statistic. The statistical comparisons were performed within the test group by dividing it into two subgroups, those with and without HP, since the control group did not receive any treatment. The differences in study variables, pre- and posttreatment were analyzed by paired samples *t* -test; the differences between HP (+) and HP (−) subgroups were compared by a Student’s *t* -test or a Mann–Whitney *U* -test, depending on whether they were distributed normally or not. Comparisons of nominal variables and percentages were made by Pearson’s Chi-square test. A *p* -value of <0.05 was considered statistically significant. The analysis was performed using the IBM SPSS, version 21.0 for Windows.

## Results

### Patient Matching

No differences were observed between the patient and control groups regarding sex (14/33 and 14/32 males, respectively, Chi-square, *p* = 0.914) or age (55.5 ± 12.4 and 55.5 ± 13.4 years, respectively, *t* -test [62.22], *p* = 0.944).

### Prevalence of *Helicobacter pylori* Infection in Patients with and without Periodontitis

HP was not detected in the saliva by PCR in any patient or control group, but it was detected in the stomach of seven controls and six patients (Chi-square: *χ* ^2^ (1) = 0.138, *p* = 0.71).

### Prevalence of *Helicobacter pylori* Infection according to Periodontitis Classification

No differences in periodontitis stages or grades were observed posttreatment. HP (+) patients presented with higher disease severity for staging (Chi-square = 11.873, *p* = 0.003) and grading (Chi-square = 7.792, *p* = 0.02; Table 1 ).

**Table 1:** Patients staging and grading pretreatment

Periodontitis classification		*Helicobacter pylori* in stomach		Total
		Yes	No	
Pretreatment stage	2	0	6	6
	3	2	19	21
	4	4	2	6
Pretreatment grade	1	0	2	2
	2	0	15	15
	3	6	10	16

### Periodontitis Treatment in Patients with or without Gastric *Helicobacter pylori* Infection

All patients with HP underwent successful eradication treatment. Measurements of pre- and posttreatment BOP, PI, PPD, and CAL in patients with and without gastric HP infection are presented in Table 2 . All patients improved with treatment, with a comparative benefit for patients who had HP (+). Benefits in CAL for both groups were the smallest, with very little benefit in HP (+) patients and no benefit for HP (−) patients. The PPD was decreased by approximately 1.3 mm in HP (+) patients and by approximately 0.9 mm in HP (−) patients.

**Table 2:** Measurements of pre- and post-treatment BOP, PI, PPD, and CAL in patients with and without gastric Helicobacter pylori infection

Gastric *H. Pylori* detection	Periodontitis clinical markers	Mean	SD	Mean difference	SD	***t*** -test (paired samples)	*p* -Value	Effect size (d)
Yes ( *n* = 6)	BOP pretreatment (p/tp)	0.814	0.224	0.674	0.203	8.112, df = 5	<0.001	3.311
	BOP posttreatment (p/tp)	0.139	0.051					
	PI pretreatment (p/tp)	0.720	0.289	0.596	0.308	4.742, df = 5	0.005	1.935
	PI posttreatment (p/tp)	0.124	0.043					
	PPD pretreatment (mm)	3.843	1.721	1.278	0.633	4.938, df = 5	0.004	2.018
	PPD posttreatment (mm)	2.565	1.109					
	CAL pretreatment (mm)	4.403	1.901	0.183	0.257	1.748, df = 5	0.141	0.712
	CAL posttreatment (mm)	4.220	1.850					
No ( *n* = 27)	BOP pretreatment (p/tp)	0.502	0.281	0.303	0.191	8.232 df = 26	<0.001	1.584
	BOP posttreatment (p/tp)	0.199	0.119					
	PI pretreatment (p/tp)	0.388	0.266	0.223	0.195	5.945, df = 26	<0.001	1.144
	PI posttreatment (p/tp)	0.164	0.119					
	PPD pretreatment (mm)	3.608	0.837	0.905	0.365	12.889, df = 26	<0.001	2.479
	PPD posttreatment (mm)	2.702	0.594					
	CAL pretreatment (mm)	4.217	1.271	-0.003	0.027	-0.723, df = 26	0.476	0.111
	CAL posttreatment (mm)	4.221	1.258					
Abbreviations: BOP, bleeding on probing; CAL, clinical attachment level; df, degrees of freedom; p/tp, points/total points; PI, plaque index; PPD, probing pocket depth; SD, standard deviation.								

### Combination of Eradication Treatment and Quadrant Scaling and Root Planning

Table 3 presents the comparisons of differences in pre- and posttreatment POB, PI, PPD, and CAL in patients with and without gastric HP infection. Periodontal debridement provided additional benefit for patients with HP who undergo treatment for the infection compared with HP (−) patients.

**Table 3:** Comparisons of differences in pre- and posttreatment POB, PI, PPD, and CAL in patients with and without gastric Helicobacter pylori infection

Periodontitis clinical markers	*H. pylori* in stomach	*n*	Mean	SD	*t* -test ( ***t*** )/Mann–Whitney ( *z* )	*p* -Value	Improvement (%)	Effect size (d)
Difference in BOP (p/tp)	Yes	6	0.674	0.203	*t* = 4.251	<0.001	82.18	1.919
	No	27	0.303	0.191			58.04	
Difference in PI (p/tp)	Yes	6	0.596	0.308	*z* = 2.474	0.013	76.72	1.714
	No	27	0.223	0.195			52.69	
Difference in PPD (mm)	Yes	6	1.278	0.633	*t* = 1.965	0.060	33.13	0.887
	No	27	0.905	0.365			24.43	
Difference in CAL (mm)	Yes	6	0.183	0.257	*z* = 2.918	0.004	6.60	0.805
	No	27	0.003	0.027			0.22	
Abbreviations: BOP, bleeding on probing; CAL, clinical attachment level; p/tp, points/total points; PI, plaque index; PPD, probing pocket depth; SD, standard deviation.								

## Discussion

The role of the oral cavity, especially when periodontitis is present, in the transmission and recurrence of HP infection is a subject of discussion and considerable divergence. In the present study, HP was not detected in the saliva of patients with periodontitis or healthy patients, regardless of gastric HP infection. The result is consistent with previous studies, in which HP was not detected in the saliva of 49 patients with dyspepsia 21 ; the subgingival plaque of 115 patients 22 ; the supra- or subgingival plaque of 62 patients with gingivitis, mild and moderate periodontitis 23 ; or the saliva, tongue, and dental plaque of 43 patients with gastric disorders. 24 A large study did not detect HP in 1,000 periodontal pockets of 336 adult patients with periodontitis, 25 while a 3.4% prevalence of HP positivity was detected in the saliva of 58 clinically healthy volunteers. 26 A recent study did not find an association between periodontitis and the presence of HP in dental plaque of 50 patients with periodontitis and 50 controls. 27 On the other hand, high prevalence of HP in saliva (55%) and dental plaque (97%) and lower ones in the stomach (26.2%) led to the opinion that HP belongs to the normal oral flora, 28 or the oral cavity serves as a reservoir for gastric HP infection. 29 Other authors argue that HP exists in the oral cavity only as a transient organism, as other competing species colonize and predominate. 30 The transient presence of HP in the oral cavity has been attributed to the contamination of the latter by gastric fluid due to reflux. 23 29 The transient presence of oral HP is supported by high immunoglobulin (Ig)-A concentrations detected in the saliva of 100 adult women with and without periodontitis, regardless of HP detection in the mouth. 31 Also, in the present study, there was no direct correlation between gastric HP infection and periodontal condition regardless of the presence of HP in the oral cavity. There is disagreement in the literature on this issue, as some researchers agree with this result 32 and some do not. 33 The comparison of all results of the studies named above presents difficulties, as they have applied different methodological procedures (primers, sampling methods, and protocols) 34 on different populations. 15 The possibility of different HP genotypes in the saliva, stomach, and stools of the same person adds to the diagnostic difficulty, 35 as PCR cross-sensitivity with other *Helicobacter* strains 34 and false-positive results from dead bacteria. 36

The prevalence of HP in the saliva is lower than those of dental plaque, regardless of the measurement method, 15 as factors, such as HP adhesion to biofilm, saliva flow, 37 and its antimicrobial content, 24 may reduce the detectable microbial load. Salivary gland secretions, viruses, fungi, and epithelial cells, 36 the changing pH, 38 and the time of sampling, due to the changing salivary flow during the day, may cause detection problems. 36

In the present study, there was 100% efficacy of gastric HP eradication, in accordance with the literature. In a meta-analysis of seven randomized controlled trials (RCTs), including 691 participants, periodontal treatment combined with eradication treatment increased the rate of gastric HP eradication compared with eradication therapy alone. 39 In another meta-analysis, eradication treatment is more effective in the recurrence of gastric HP, combined with periodontal debridement than alone. 40 Gastric HP eradication occurred at a lower rate compared with the present study (87.4 vs. 100%), 3 months after triple treatment without periodontal therapy, in patients who were HP (+) in the stomach and HP (−) in the oral cavity. 41

Although the present study did not find an association between gastric HP infection and periodontitis, it showed an association between gastric HP infection and the severity of periodontal disease, regardless of HP detection in saliva. HP was not detected in the stomach of patients with stage-2 periodontitis, while the prevalence was 9.5 and 66.6% for stages 3 and 4, respectively. Scarce evidence is available on this association, as most research focuses on the association between periodontitis independently of its severity and gastric HP infection. In a recent study, 39.4% of patients with mild periodontitis were diagnosed with HP infection, while this prevalence rose to 70 and 85.7% in patients with moderate and advanced periodontitis, respectively. 42 Another study 43 found a positive association between gastric HP infection and the number of lost teeth as a proxy of advanced periodontal disease. The detection of HP only in moderate-to-severe periodontal pockets 16 supported this association. Adachi et al measured lactate dehydrogenase (LDH) and hemoglobin (Hb) concentrations in saliva to diagnose the degree of periodontal tissue damage and the degree of bleeding gums, respectively, in 686 patients, and showed that gastric HP infection is a potential risk factor for the onset and deterioration of periodontal disease. 44

The association between gastric HP infection and advanced grade of periodontal disease is another finding of the present study, as all HP (+) patients grade to the rapid rate of periodontitis progression (grade C). As the grades “reﬂect biologic features of the disease including evidence of, or risk for, rapid progression, anticipated treatment response, and effects on systemic health,” 17 this finding could partly explain the improvement and the high-to-moderate effect sizes of HP (+) patients who received eradication in addition to periodontal treatment. As the present study did not attempt to detect HP in other parts of the oral cavity, where the microorganism could be present, antibiotic treatment may have helped eliminate the hidden HP that could potentially cause rapid progression and/or poor response of periodontitis to previous treatments. Thus, while all patients benefited from QSRP, those receiving adjuvant HP eradication treatment showed an improvement in most periodontal clinical markers (BOP, PI, and CAL) compared with those HP (−) patients who did not receive eradication treatment. Perhaps, the relatively small sample size did not allow PPD to achieve statistical significance. However, according to much of the literature, greater clinical improvement is reported for patients with periodontitis, receiving both periodontal treatment and systemic antibiotics compared with those who receive periodontal treatment alone, regardless of the presence of HP. 45 Thus, the lack of a group of HP (+) patients with periodontal disease who received only periodontal treatment without antibiotic therapy constitutes a limitation of this study, given the complexity of the pathogenic mechanisms involved in periodontal disease, it cannot be claimed that the further improvement of the periodontal status is a direct result of the medication.

As HP was not detected at root canals of teeth with asymptomatic apical periodontitis and aspirates from acute apical abscesses, it seems that the necrotic root canal does not serve as an HP reservoir. 46 On the contrary, it seems that HP infection may be indirectly related to periodontitis, even its advanced forms, through periodontal disease bacteria only, such as *Campylobacter rectus* and *Tannerella forsythia* , 30 47 48 which can compete and bind HP strains. This binding, which might be the reason for the temporality of HP in the mouth, may lead to a cross-antigenicity of HP and periopathogens through heat-shock proteins, resulting in increased inflammatory immune response in the stomach and the mouth. 30 47

Associating gastric HP infection with the stages and degrees of periodontitis, rather than periodontitis as a dichotomous variable, is an advantage of the present study. However, the small sample size and the study type (single center) constitute disadvantages. Further research is needed on larger populations of patients and controls to draw safe conclusions.

## Conclusion

In conclusion, the present study indicates that periodontitis is not directly related to gastric HP infection, neither is saliva a supply reservoir of gastric HP. However, there is an association of HP gastric infection with advanced stages and grades of periodontitis when these two conditions coexist. Although the periodontal clinical markers improved after QSRP in all patients, regardless of HP gastric infection, BOP, PI, and CAL improved more in patients who underwent HP eradication compared with those who underwent QSRP only; thus, periodontal evaluation and treatment combined with HP eradication in gastric HP (+) patients are recommended.
